# Predicting and Improving Interlaminar Bonding Uniformity during the Robotic Fiber Steering Process

**DOI:** 10.3390/polym15010019

**Published:** 2022-12-21

**Authors:** Pan Zhao, Bijan Shirinzadeh, Xiaodong He, Jian Guo, Kaining Shi, Biyao Qiang, Qichao Jin, Fengping Li

**Affiliations:** 1 School of Intelligent Manufacturing and Control Technology, Xi’an Mingde Institute of Technology, Xi’an 710124, China; 2 School of Mechanical Engineering, Northwestern Polytechnical University, Xi’an 710072, China; 3 Robotics and Mechatronics Research Laboratory, Department of Mechanical and Aerospace Engineering, Monash University, Clayton, Melbourne 3800, Australia; 4 School of Mechanical Engineering, Inner Mongolia University of Technology, Hohhot 010051, China; 5 College of Mechanical and Electrical Engineering, Wenzhou University, Wenzhou 325035, China; 6 Key Laboratory of Road Construction Technology and Equipment of MOE, Chang’an University, Xi’an 710061, China; 7 Institute of Laser and Optoelectronics Intelligent Manufacturing, Wenzhou University, Wenzhou 325000, China

**Keywords:** robotic fiber steering process, fiber waviness, interlaminar bonding uniformity

## Abstract

With their high specific stiffness, corrosion resistance and other characteristics, especially their outstanding performance in product weight loss, fiber-reinforced resin matrix composites are widely used in the aviation, shipbuilding and automotive fields. The difficulties in minimizing defects are an important factor in the high cost of composite material component fabrication. Fiber steering is one of the typical means of producing composite parts with increased strength or stiffness. However, fiber waviness is an important defect induced by fiber steering during the fiber placement process. Meanwhile, the laying speeds of the inner and outer tows along the path width direction are different during the fiber steering process, resulting in different interlaminar bond strengths. Therefore, the fiber waviness and uneven interlaminar bonding strength during fiber steering not only affect the dimensions of a composite product, but also influence the mechanical properties of the part. This study aims to reduce fiber waviness and improve interlaminar bonding uniformity along the path width direction using a multi-piece compaction roller. By analyzing the mechanism of the generation of fiber waviness, the interlaminar bonding strength for each tow during fiber steering is investigated. Through analyzing and optimizing the compaction force, laying temperature and laying velocity during fiber steering experiments, the optimization approach is verified.

## 1. Introduction

Fiber-reinforced polymer composites offer the potential for the development of products with high strength, a high modulus and reduced weight, and are thus suitable for manufacturing components in several industries [1,2,3]. However, the high processing cost hinders composite materials being widely used [4]. Winding technology and automated placement technology are common technologies for fabricating composite products with low cost [5,6,7,8]. As a key branch of low-cost composite processing technologies, robotic fiber placement (RFP) technology provides several advantages for the fabrication of composite components with complex surfaces, including real-time process parameter control [9], low rejection rates and large layup angles [10].

Due to the benefits of RFP, several prepreg tows can be laid simultaneously onto the surface of the substrate, and this approach has been applied to producing components with complex structures. For unidirectional prepreg fibers, the maximum strength is along the fiber direction. Through steering the fibers to match the load path of the part, the strength of the structure can be improved, and the dependence on the ultimate strength of the resin can be reduced. Thus, fiber steering is one of the typical means of increasing the strength or stiffness of the composite parts. During the steering process, the prepreg tow can be deformed due to the tensile modulus of the reinforced fiber and the viscoelasticity of the resin. A large number of defects will occur while laying surfaces with a large curvature, such as gaps and fiber waviness [11,12]. Fiber waviness is one of the major influences on the mechanical performance of composites [13,14,15]. It significantly affects mechanical properties such as stiffness, strength and fatigue, and dramatically reduces the load-carrying capacity of the material [16]. Velmurugan et al. investigated the influence of fiber waviness on the Young’s moduli, the shear moduli and Poisson’s ratios of unidirectional discontinuous fiber-reinforced composites [17]. Zhao et al. calculated the elastic constants and tensile strength of unidirectional laminates with fiber waviness. Experimental results showed that the tensile properties infade dramatically with increasing magnitude of the waviness [18]. Sitohang et al. demonstrated in their experiments that waviness severity affects stress at first failure and compressive damage development [19]. Wang et al. manufactured and tested specially induced fiber waviness defects, and their experimental results showed a maximum of a 58.1% drop in compressive failure load for the most severe level [20]. In summary, fiber waviness directly affects the quality of the composite product during the fiber placement process.

Meanwhile, different laying speeds of the inner and outer tows along the path width direction will occur during the fiber steering process, which results in different interlaminar bond strengths. Interlaminar bonding strength is an important indicator for the quality of composite laminates. Hence, uneven interlaminar bonding strength during fiber steering will influence the mechanical properties of the composite components. Aized and Shirinzadeh optimized the robotic fiber placement process using the response surface method [21]. The process parameters were analyzed, including compaction force, laying temperature and laying speed. Song et al. established models for predicting the relationship between the bonding strength between layers in the forming process parameters, and optimized the in situ forming process parameters [22]. Zhang et al. investigated the effects of processing parameters on the interlaminar strength of CF/PEEK laminates, and showed that the main mechanism of mechanical property loss for low melt viscosity matrices in the laminates was due to flow or extrusion of the matrix during processing [23]. In short, the interlaminar bonding strength can be improved by optimizing the fiber placement process factors, such as compaction force, laying velocity, laying temperature, etc.

In this study, a method for fabricating a composite laminate with optimized homogeneity in the tow width direction is proposed. It aims to decrease the fiber waviness during the steering of the prepreg tows, and to improve the interlaminar bonding uniformity in the tow width direction during the fabrication of components with large curvature. The structure of this paper is as follows: Section 2 provides a theoretical analysis of the interlaminar bonding process. The mechanism of fiber waviness generation is investigated, and the method of improving the interlaminar bonding uniformity in the width direction is outlined in Section 3. Section 4 describes the experiments for improving the interlaminar bonding uniformity for each tow. Section 5 gives the conclusions and future research directions.

## 2. Analysis of Interlaminar Bonding Process

**Figure 1:**
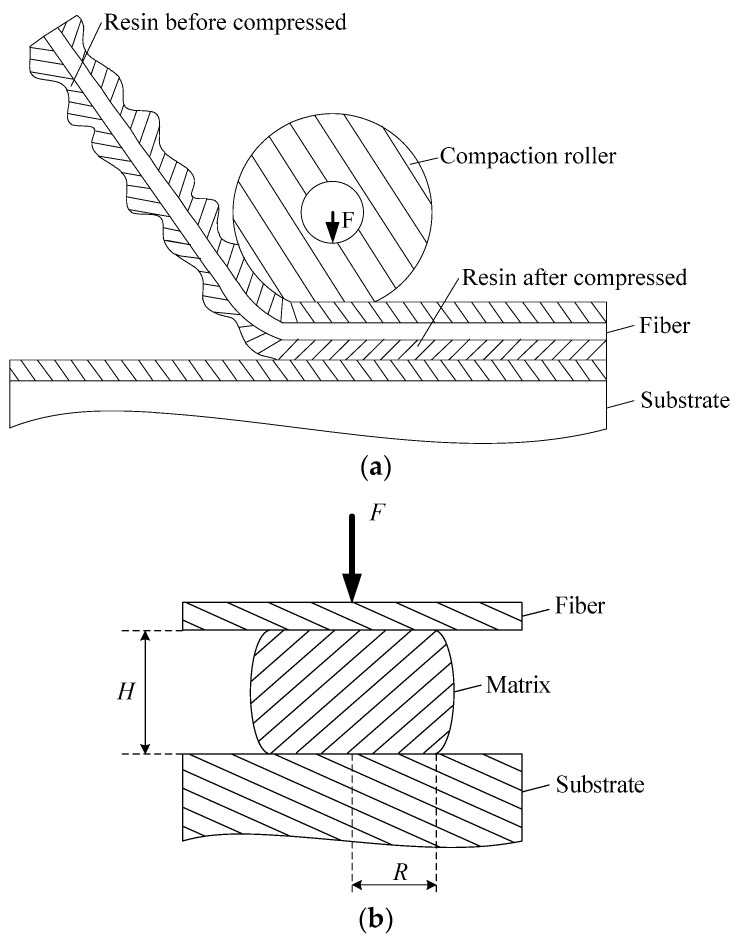
Squeezing flow of resin patch under parallel plate. (**a**) Interface between prepreg tow and the substrate surface. (**b**) The contact surface of the thermoset prepreg tow.

Gutowski and Bonhomme modeled the resin on the surface of the prepreg as resin adhesive sheets, and considered that the bonding between the prepreg and the mold was, in fact, a process in which the resin adhesive sheet was grown to infiltrate the interlayer [24]. Ignoring the penetration of the resin in the prepreg fiber, the growth of the resin adhesive sheet could be regarded as the extrusion of the resin between the parallel plates, where the length of the parallel plate is the contact length $L_{c}$ between the compaction roller and the prepreg. The squeezing flow of the resin patch between the prepreg tow and the substrate surface of the thermoset RFP process is shown in Figure 1.

The extrusion model of the resin between the parallel plates is given by the Scott equation [25]:

(1) $$F=m\pi \frac{{\left(2+s\right)}^{n}}{\left(3+n\right)}\cdot \frac{{\left(-H^{\prime }\right)}^{n}R^{3+n}}{2^{n}H^{\left(1+2n\right)}}$$

where F is the compaction force, m and n are the fluid power-law model coefficients, s=1/n, H is the height of the resin, $H^{\prime }=dH/dt$, and R is the infiltrated radius of the resin.

Although most of the resins are pseudoplastic fluids, Gutowski and Bonhomme found that modeling the resin as a Newtonian fluid could still accurately describe the flow of the resin between the interface layers [24]. For Newtonian fluids, the Scott equation can be written as follows.

(2) $$F=\frac{3\pi \mu }{8}\cdot \frac{\left(-H^{\prime }\right)R^{4}}{H^{3}}$$

where *μ* is the Newtonian fluid viscosity of the resin. Assuming the volume of the fluid remains unchanged during extrusion, the fluid volume can be expressed by the following equations:

(3) $$V=\pi HR^{2}=A\left(0\right)H\left(0\right)$$

(4) $$R^{2}=\frac{A\left(0\right)H\left(0\right)}{\pi H}$$

where H(0) is the initial fluid height, and A(0) is the initial infiltrated area. Substituting Equation (4) into Equation (2), the expression for the compaction force F is given as follows:

(5) $$F=-\frac{3\mu }{8\pi }{\left(A\left(0\right)H\left(0\right)\right)}^{2}\frac{H^{\prime }}{H^{5}}$$

**Figure 2:**
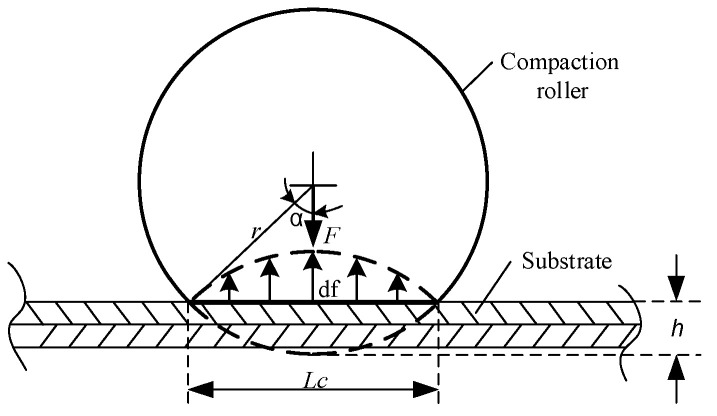
The contact area between the compaction roller and the substrate.

During the RFP process, the compaction force is provided by the compaction roller on the surface of the prepreg tows. Due to the rubber roller deformation, the contact area between the compaction roller and the substrate is shown in Figure 2. 

**Figure 3:**
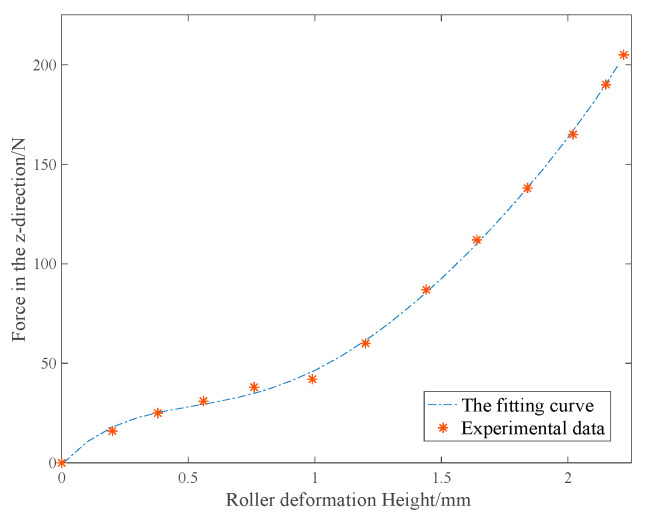
Relationship between vertical deformation height and compaction force.

The relationship between the roller vertical deformed height h and the compaction force F is shown in Figure 3. The contact length $L_{c}$ can be calculated as follows:

(6) $$\cos\alpha =\frac{r-h}{r}$$

(7) $$L_{c}=2r\sin\alpha =2r\sqrt{1-{\left(\cos\alpha \right)}^{2}}=2\sqrt{2rh-h^{2}}$$

(8) $$\int_{0}^{L_{c}}f\;dl=F$$

where α is the angle between the compaction force direction and the force affected area boundary of the compaction roller, and r is the radius of the compaction roller. The time $t_{c}$ of compaction force can be calculated as follows [26]:

(9) $$t_{c}=\frac{L_{c}}{V}$$

where V is the laying velocity, and $L_{c}$ is contact length between compaction roller and substrate. Considering the time-of-compaction force effect, Equation (5) can be modified as follows:

(10) $$\int_{0}^{t_{c}}Fdt=-\frac{3\mu }{8\pi }{\left(A\left(0\right)H\left(0\right)\right)}^{2}\int_{H\left(0\right)}^{H\left(t_{c}\right)}\frac{1}{H^{5}}dH$$

Upon integrating the left and right sides of Equation (10), and combining Equation (9), the following expression can be obtained:

(11) $$F\frac{L_{c}}{V}=\frac{3\mu }{32\pi }{\left(A\left(0\right)H\left(0\right)\right)}^{2}\left(\frac{1}{H^{4}\left(t_{c}\right)}-\frac{1}{H^{4}\left(0\right)}\right)$$

where $H\left(t_{c}\right)$ is the fluid height after experiencing the compaction force. The interlaminar bonding strength is represented by the interlaminar contact degree $D_{c}$, which can be expressed by the ratio of the initial fluid height and the fluid height after experiencing compaction force. Based on Equation (11), and combined with the previous assumptions of the fluid volume remaining unchanged during extrusion, the interlaminar contact degree can be obtained as follows:

(12) $$D_{c}=\frac{A\left(t_{c}\right)}{A\left(0\right)}=\frac{H\left(0\right)}{H\left(t_{c}\right)}={\left[1+\frac{32\pi }{3}{\left(\frac{H\left(0\right)}{A\left(0\right)}\right)}^{2}F\frac{L_{c}}{V\mu }\right]}^{\frac{1}{4}}$$

where $A\left(t_{c}\right)$ is the infiltrated area after experiencing the compaction force.

In addition to the resin characteristics and compaction roller characteristics, the contact degree will be influenced by the process parameters. When the laying speed increases, the time in which the compaction pressure takes effect decreases. Thus, the prepreg tows could not be bonded onto the surface of the substrate laminate completely. Furthermore, the interlaminar bonding strength will decrease. When the laying temperature increases, the resin viscosity decreases. As a result, the infiltrated effect of the resin on the contact surface becomes better, increasing the interlaminar bonding strength. When the compaction force increases, the contact area between the compaction roller and the substrate increases. The trapped air at the interface can be ejected by the compaction roller more easily than before. As a consequence, the interlaminar bonding strength will increase. In summary, the compaction force, laying temperature and laying velocity will directly change the contact degree.

## 3. Analysis of Fiber Waviness and the Interlaminar Bonding Strength for Each Tow

### 3.1. The Mechanism of the Generation of Fiber Waviness 

**Figure 4:**
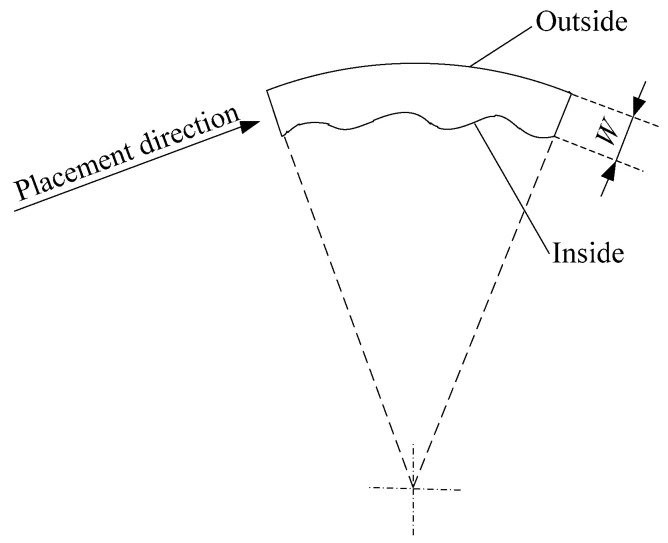
A schematic diagram of the generation of fiber waviness.

During the RFP process, the laying path of the fiber placement is along the centerline of the prepreg tows in the width direction. Assuming that the prepreg tow is a beam with thickness c and width W, the width of the prepreg tow is much greater than the thickness. Based on beam bending theory [27,28], the stress in the width direction is dramatically larger than in the thickness direction. The deformation of the prepreg tows in the actual process is shown in Figure 4. During the fiber steering process, the inside part of the prepreg tow is under compression and the outside part is under tension. Due to the anisotropic nature of the unidirectional prepreg tow, the uncured prepreg tow only has a high tensile modulus in the fiber direction. Therefore, the tensile stress is small, and is not enough to destroy the fibers on the outside during the fiber steering process. Because the compaction roller will be in close contact with the prepreg tows, the fibers on the outside do not tilt in the normal direction, and only slide to the inside along the width direction. At the same time, the fibers inside will buckle. So, uneven fiber internal and external stress is one of the factors causing fiber waviness.

Another reason for the occurrence of fiber waviness is the uneven internal and external laying speeds while the roller lays the circle. Figure 4 shows the path of the roller laying the circle. In fact, the compaction time of each tow is the same, but the length of each tow’s laying path is different. Hence, the velocity of each tow is different. The velocity will increase from the inner to the outer curvature. Because each tow has the same tensile modulus, the inner tow will be deformed easily using the one-piece compaction roller.

### 3.2. The Multi-Piece Compaction Roller

**Figure 5:**
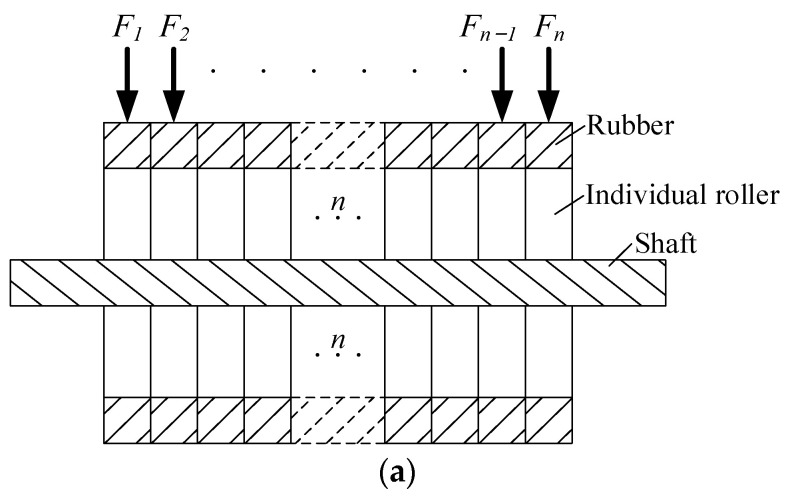

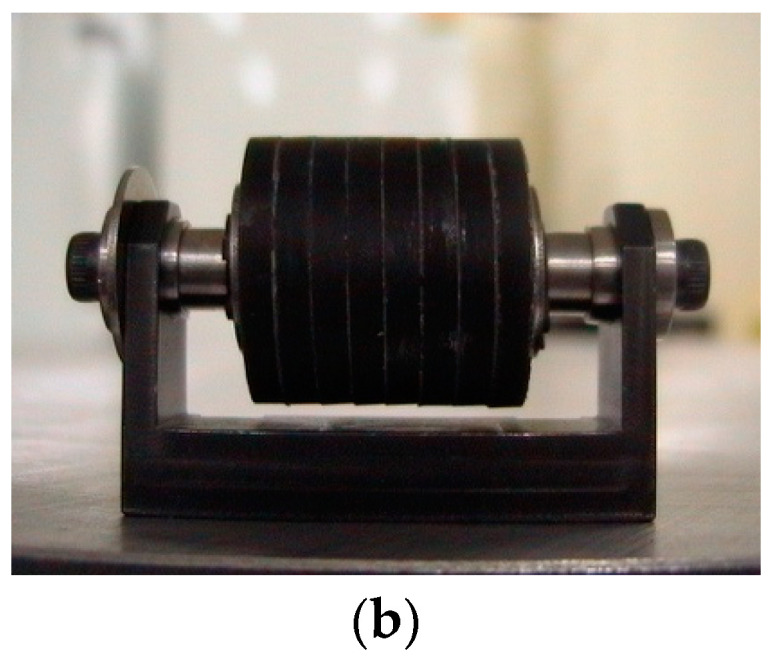
The multi-piece compaction roller. (**a**) The optimized compaction roller with n individual roller components. (**b**) The multi-piece compaction roller for the experiment.

Due to the analysis above, the normally used one-piece compaction roller is not adequate for the fiber steering placement process. The compaction roller needs to be improved to decrease the fiber waviness for fiber steering experiments. The multi-piece roller is shown in Figure 5.

This optimized multi-piece compaction roller has n individual roller components for the application of n tows that can be placed by the robot simultaneously, and these have to be separated to enable different rotation velocity for each roller component. If the component can rotate separately, then the outer component can rotate faster than the inner component during the laying of a curved path. After using the multi-piece compaction roller, the amount of fiber waviness can be decreased.

### 3.3. Analysis of the Interlaminar Bonding Strength for Each Tow

**Figure 6:**
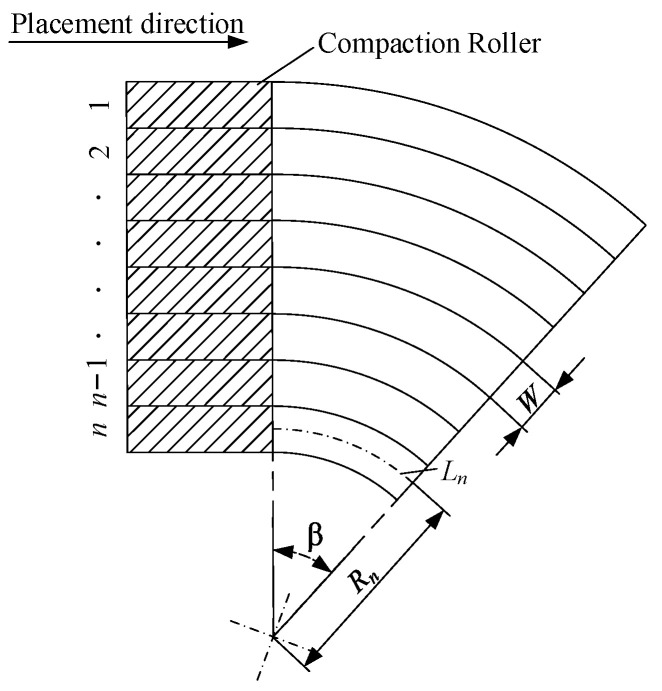
The path of laying the circle.

After using the multi-piece compaction roller, the laying velocity of each tow can be modified. Even though the fiber waviness can be decreased using the multi-piece compaction roller, the interlaminar bonding strengths for each tow from the inner to the outer tow are still different. In Equation (12), the interlaminar bonding strength is affected by three main parameters, including the compaction force, the laying velocity and the laying temperature. The laying velocity of each tow from outside to inside during the fiber steering process is different, and the interlaminar bonding strength can be maintained for each tow by modifying the other process parameters. Because the width of the prepreg tow is quite small, it is difficult to control laying temperatures that are different for two adjacent tows. The best choice is to modify the compaction force to specify the variation rule of the laying velocity. The multi-piece compaction roller can supply different compaction forces to different pieces of the roller, which is shown in Figure 5a. Figure 6 shows the laying path for the fiber steering circle.

Using the multi-piece roller for fiber steering, as shown in Figure 6, the path length of the *i*-th tow can be expressed as follows:

(13) Li=2π(Ri−(i−1)∗W)∗β2π , (i=1, 2, … , n )

Setting the first tow’s laying speed at $V_{1}$, the fiber steering process time t can be written as:

(14) t=L1V1

The fiber steering process time for every tow is the same, and the laying speed of the *i*-th tow can be calculated as:

(15) Vi=Lit

The interlaminar bonding strength for the *i*-th tow is given as follows:

(16) $$D_{c_{i}}={\left[1+\frac{32\pi }{3}{\left(\frac{H\left(0\right)}{A\left(0\right)}\right)}^{2}\frac{F_{i}\cdot L_{c_{i}}}{V_{i}\cdot \mu }\right]}^{\frac{1}{4}}$$

In order to obtain an even interlaminar bonding degree for each tow in the width direction, the following constraint needs to be enforced:

(17) $$D_{c_{1}}=D_{c_{2}}=\cdots =D_{c_{n}}$$

(18) $$\frac{F_{1}\cdot L_{c_{1}}}{V_{1}}=\frac{F_{2}\cdot L_{c_{2}}}{V_{2}}=\cdots =\frac{F_{n}\cdot L_{c_{n}}}{V_{n}}$$

Finally, the modified compaction force for each tow can be calculated as follows:

(19) $$F_{i}=\frac{F_{1}\cdot L_{c_{1}}\cdot V_{i}}{V_{1}\cdot L_{c_{i}}}$$

Based on Figure 6 and the equations above, the interlaminar bonding degree of each tow can be kept similar during the fiber steering process by modifying the compaction force and the contact length.

## 4. Testing

### 4.1. RFP System and Material Preparation

**Figure 7:**
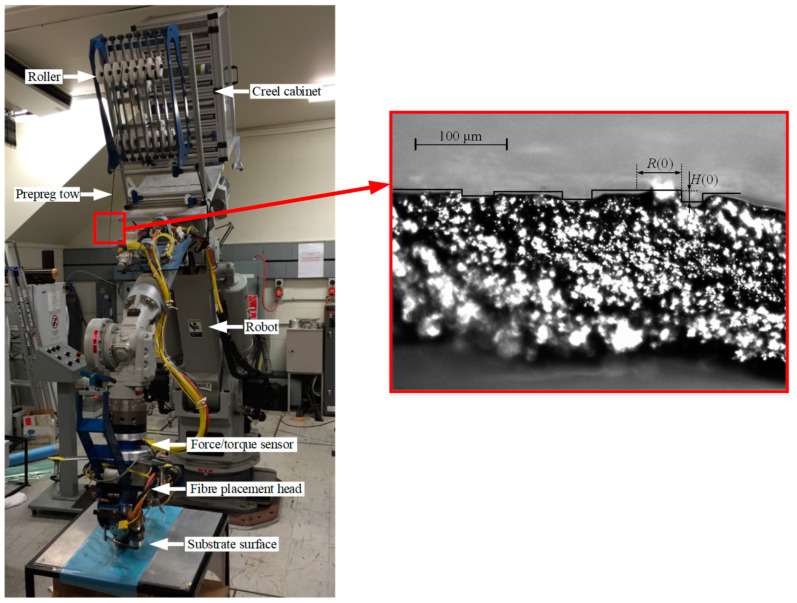
The RFP system and cross-sectional photomicrograph of prepreg tow.

The robotic fiber placement research device and the carbon fiber/epoxy prepreg tow are shown in Figure 7. The initial resin height H(0), and the initial infiltrated radius of the resin R(0) can be measured using a Nikon ECLIPSE E200 optical microscope (Manufactured by Nanjing Nikon Jiangnan Optical Instrument Co., Ltd. in Nanjing, China.), and the details of the composite material are shown in Table 1 [29].

### 4.2. Experiment for Decreasing the Fiber Waviness

**Figure 8:**
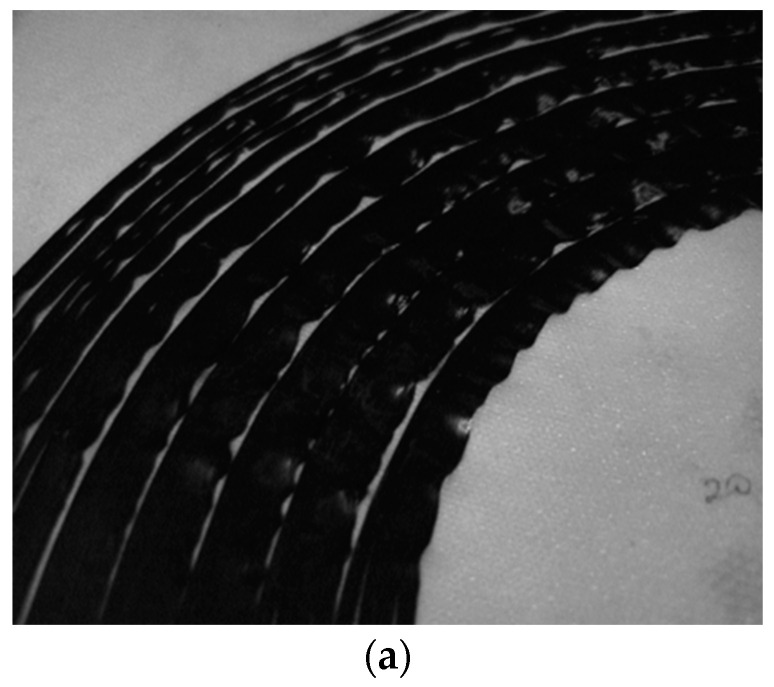

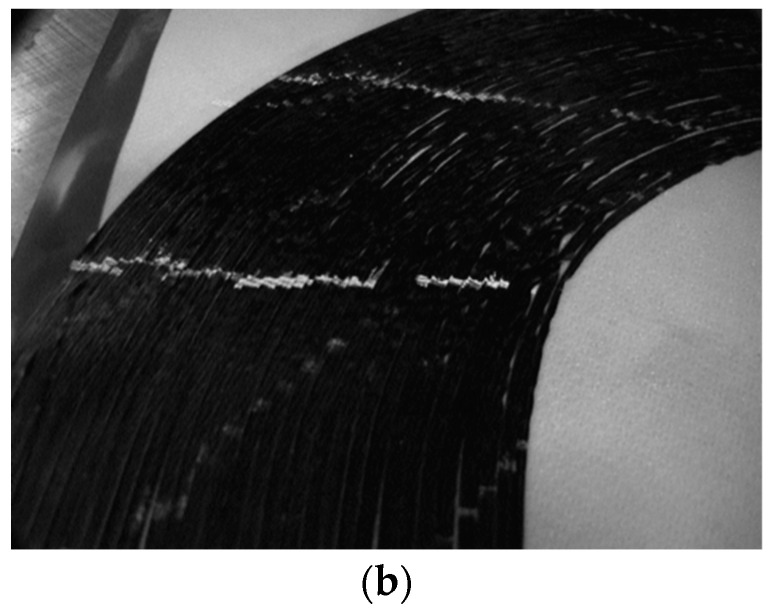
The laying circle using one-piece and multi-piece compaction rollers. (**a**) Laying circle using one-piece compaction roller. (**b**) Laying circle using multi-piece compaction roller.

Experiments were performed that involved a fabricated specimen in order to analyze the impact on the fiber waviness using the optimized compaction roller. The specimen for the analysis was a fiber steering quarter circle, which used a one-piece compaction roller and a multi-piece compaction roller for comparison. The experimental ambient temperature was (20 ± 2) °C and the humidity was (30 ± 3)%. The process parameters were set as follows: the laying velocity was 100 mm/s, the laying temperature was 100 °C and the compaction force was 20 N. The radius of the quarter-circle laying path was set to 100 mm, 112.7 mm, 125.4 mm, 138.1 mm, 150.8 mm, 163.5 mm, 176.2 mm and 188.9 mm. The laying results using the two different rollers are shown as Figure 8.

**Figure 9:**
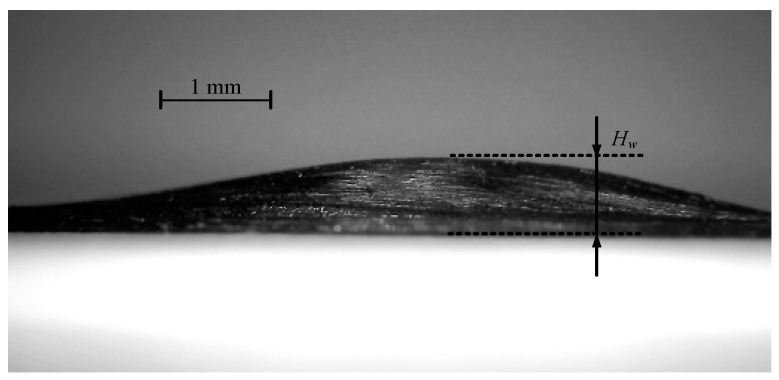
The height of the fiber waviness.

In Figure 8a, the fiber waviness occurs on the inside part of the tows. In Figure 8b, the fiber waviness is decreased by the multi-piece compaction roller. The degree of the decrease in fiber waviness during the fiber steering process can be quantified by the fiber waviness height. The height of the fiber waviness is shown in Figure 9, measured using the Nikon ECLIPSE E200 optical microscope. 

Because the specimen shown in Figure 8 lays four tows together during one placement, the highest fiber waviness height *H_W_* will occur on the inner side of the tow. The capital fiber waviness height using two different rollers is shown as Table 2, where *H_W_*_1_ is the waviness height using the one-piece roller, and *H_W_*_2_ is the waviness height using the multi-piece roller.

As seen in Figure 8b and Table 2, the fiber waviness is significantly decreased using the multi-piece compaction roller. 

### 4.3. Experiment for Improving the Interlaminar Bonding Uniformity for Each Tow

**Figure 10:**
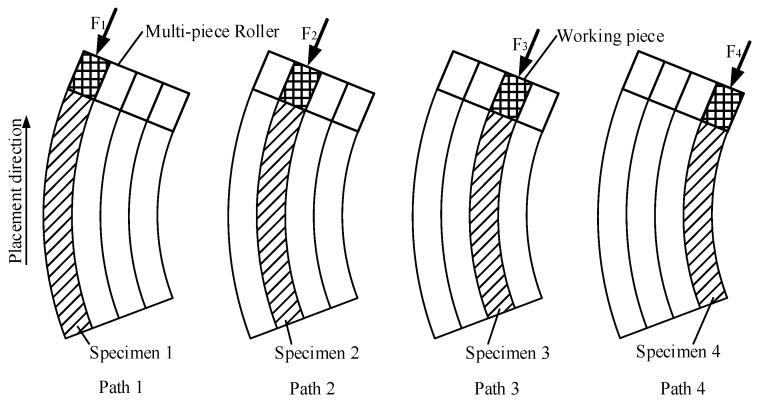
A schematic diagram of the laying paths for producing specimens.

The specimen was a one-tow/two-layer laminate using fiber steering placement. In the experiment, we used the following steps to make the sample and analyze the influence on the bonding uniformity between the layers of each tow. The experimental ambient temperature was kept the same as in the previous experiment. Except the compaction force, all the process parameters were kept constant, including the laying temperature and the laying velocity. Although the multi-piece compaction roller for the experiment can allow separate laying velocities for each tow, it cannot supply different compaction forces to different tows. In order to perform the experiments for analyzing the interlaminar bonding degree of each tow, every tow specimen was laid up using four paths (No.1 to No.4) during the placement process. The calculated compaction force was supplied to every placement process. A schematic diagram of the laying paths for producing specimens is shown in Figure 10.

**Figure 11:**
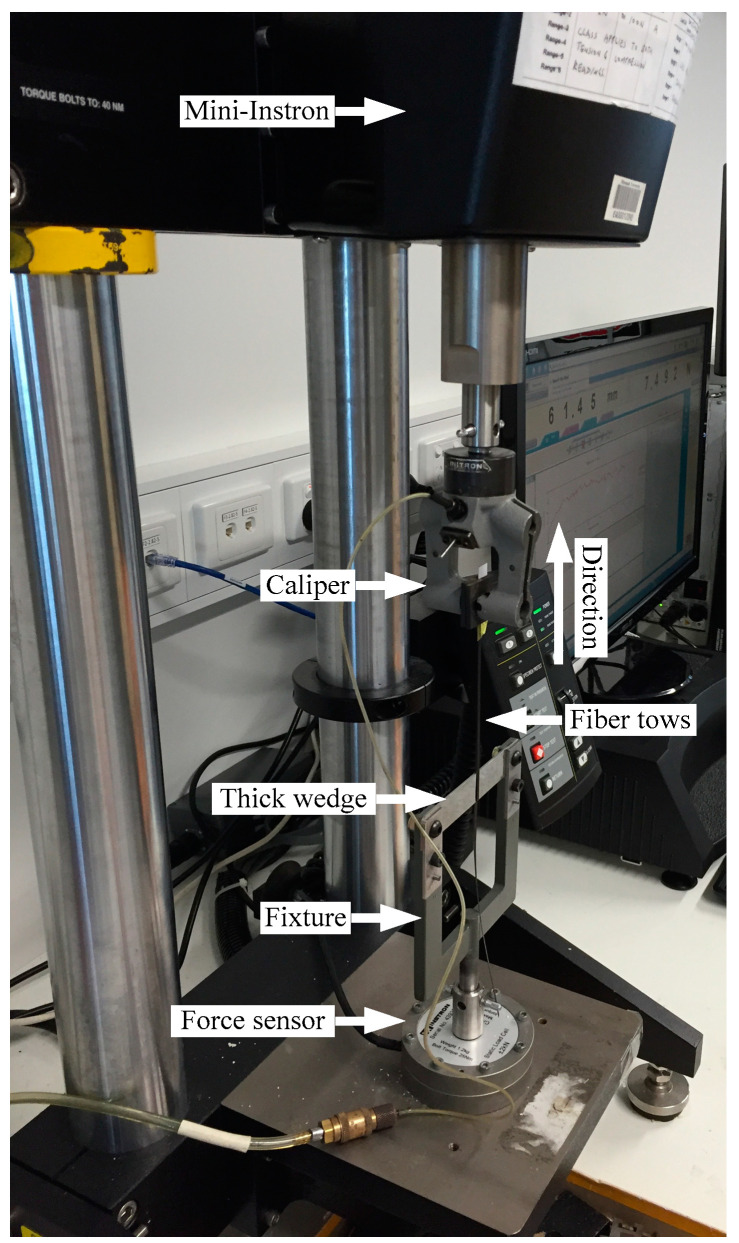
The platform for the peeling test.

Each specimen was peeled using a test piece, with the peeling process performed on a peeling test platform, to characterize the degree of interlayer bonding at the two-layer interface [30]. As shown in Figure 11, the peeling test platform consists of a Mini-Instron 5848 (Manufactured by INSTRON in Boston, U.S.A.) with 200 N units and a designed fixture. The two combined tows were peeled to produce 1.5 mm thick wedge at a speed of 50 mm/min.

**Figure 12:**
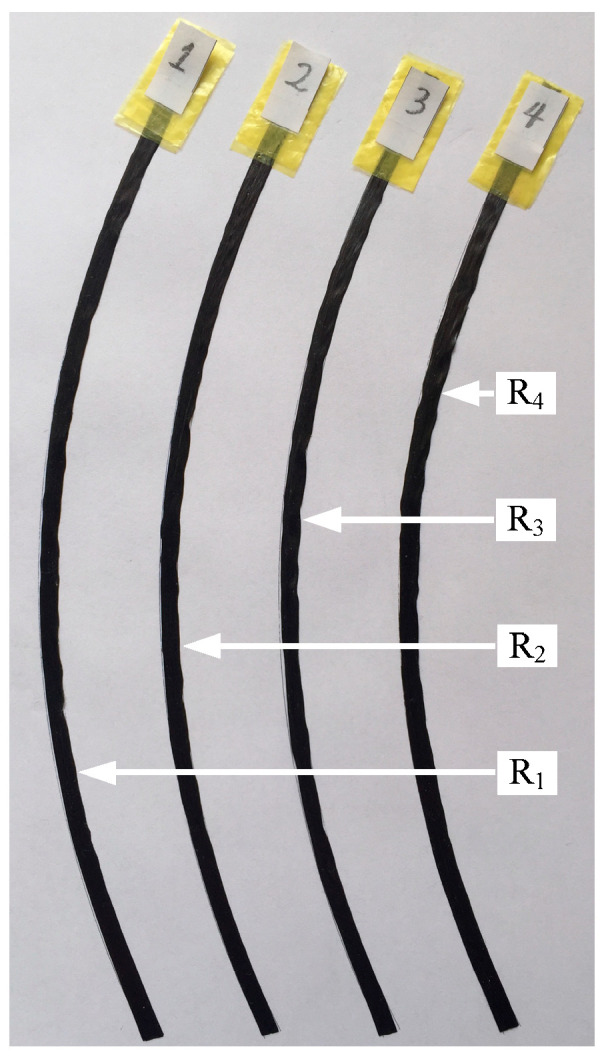
The 2 layers of test pieces.

The process parameters were set as follows: the laying velocity $V_{1}$ was 100 mm/s, the laying temperature was 100 °C and the radius of the quarter-circle laying path was 200 mm. Through Equations (16) to (18), the compaction force for each specimen was calculated, as shown in Table 3. The specimens are shown in Figure 12.

**Figure 13:**
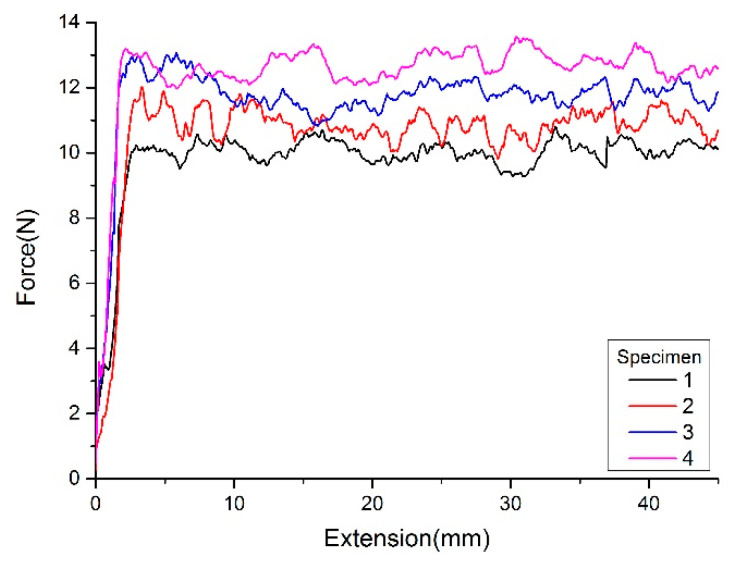
Peeling force with unchanged compaction force.

**Figure 14:**
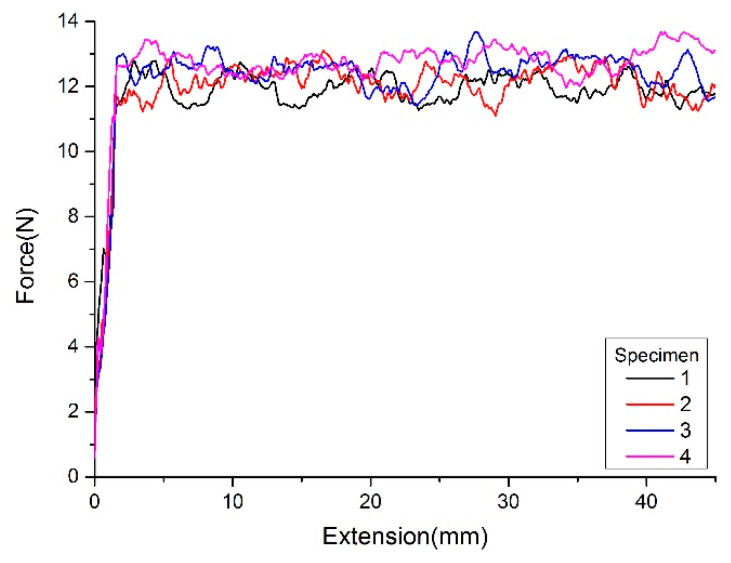
Peeling force with calculated compaction force.

Figure 13 shows each specimen’s peeling force using the unchanged 20 N compressive force. Figure 14 shows each specimen’s peeling force using the calculated compressive force. The average peeling force for specimens with the same/calculated compaction force is shown in Table 4. As shown in the test results, after optimizing the compaction force for each tow, the interlaminar bonding uniformity is higher than that of the test piece with unchanged compaction force.

## 5. Conclusions

Fiber waviness is a key defect generated during the fiber steering process and will affect product quality, especially for parts with the a large curvature. Based on a theoretical analysis of the fiber steering process, two reasons for the generation of fiber waviness were revealed, including uneven fiber internal and external stress, and uneven internal and external laying speeds. Thus, the optimized multi-piece compaction roller, with the ability to supply different laying speeds for different tows, was used to decrease this defect.

Meanwhile, uneven interlaminar bonding strength along the path width direction was caused by the different laying speeds of the inner and outer tows during the fiber steering process. For the interlaminar bonding process, the interlaminar bonding degree was influenced by the process parameters, including compaction force, laying temperature and laying velocity. Following the principle of equal bonding degree along the width direction, the interlaminar bonding uniformity during the robotic fiber steering process was improved by optimizing the compaction force for each tow.

In addition, the number of tows currently used for the fiber placement process may be up to 32 [31]. For the RFP fiber steering process with more tows laid at one time, the improvements will be more significant using the optimized method. In future research, the structure of an optimized multi-piece compaction roller that could individually supply different compaction forces for each tow will be designed, fabricated and tested.

## Figures and Tables

**Table 1:** The details of the carbon fiber/epoxy prepreg tow.

Width (mm)	Thickness (mm)	Fiber Volume	Geometric Ratio	Initial Resin Height H(0) (μm)	Initial Infiltrated Radius of the Resin R(0) (μm)
3.175	0.125	40%	0.35	150.8	55.3

**Table 2:** The fiber waviness height using two different rollers.

No.	1	2	3	4	5	6	7	8
Radius /mm	100	112.7	125.4	138.1	150.8	163.5	176.2	188.9
*H_W*1*_* /mm	2.93	2.66	2.35	2.09	1.83	1.67	1.39	1.18
*H_W*2*_* /mm	1.42	1.37	1.23	1.03	0.96	0.78	0.59	0.31

**Table 3:** Calculated compaction force for each specimen.

No.	1	2	3	4
F	20	19.7	19.2	18.8

**Table 4:** The average peeling forces.

Specimen No.	1	2	3	4
The specimen with the same compaction force	10.07	10.73	11.6	12.42
The specimen with calculated compaction force	11.76	11.93	12.26	12.57

## Data Availability

Not applicable.
